# Low Diversity and High Genetic Structure for *Platonia insignis* Mart., an Endangered Fruit Tree Species

**DOI:** 10.3390/plants13071033

**Published:** 2024-04-06

**Authors:** Caroline Bertocco Garcia, Allison Vieira da Silva, Igor Araújo Santos de Carvalho, Wellington Ferreira do Nascimento, Santiago Linorio Ferreyra Ramos, Doriane Picanço Rodrigues, Maria Imaculada Zucchi, Flaviane Malaquias Costa, Alessandro Alves-Pereira, Carlos Eduardo de Araújo Batista, Dario Dantas Amaral, Elizabeth Ann Veasey

**Affiliations:** 1 Genetics Department, Luiz de Queiroz College of Agriculture, University of São Paulo, Piracicaba 13418-900, SP, Brazil; 2 Science Center of Chapadinha, Federal University of Maranhão, Chapadinha 65500-000, MA, Brazil; 3 Exact Sciences and Technology Institute, Federal University of Amazonas, Itacoatiara 69103-128, AM, Brazil; 4 Genetics Department, Federal University of Amazonas, Manaus 69067-005, AM, Brazil; 5 São Paulo Agency for Agribusiness and Technology, Piracicaba 13400-970, SP, Brazil; 6 Museu Paraense Emílio Goeldi, Belém 66040-170, PA, Brazil

**Keywords:** Amazonian fruits, *bacurizeiro* trees, conservation genetics, genotyping-by-sequencing, SNPs

## Abstract

*Platonia insignis* is a fruit tree native to Brazil of increasing economic importance, with its pulp trading among the highest market values. This study aimed to evaluate the structure and genomic diversity of *P. insignis* (*bacurizeiro*) accessions from six locations in the Brazilian States of Roraima, Amazonas, Pará (Amazon biome), and Maranhão (Cerrado biome). A total of 2031 SNP markers were obtained using genotyping-by-sequencing (GBS), from which 625 outlier SNPs were identified. High genetic structure was observed, with most of the genetic variability (59%) concentrated among locations, mainly between biomes (Amazon and Cerrado). A positive and significant correlation (r = 0.85; *p* < 0.005) was detected between genetic and geographic distances, indicating isolation by distance. The highest genetic diversity was observed for the location in the Cerrado biome (*H_E_* = 0.1746; *H_O_* = 0.2078). The locations in the Amazon biome showed low genetic diversity indexes with significant levels of inbreeding. The advance of urban areas, events of burning, and expansion of agricultural activities are most probably the main factors for the genetic diversity reduction of *P. insignis*. Approaches to functional analysis showed that most of the outlier loci found may be related to genes involved in cellular and metabolic processes.

## 1. Introduction

Tropical fruits are increasingly gaining importance due to their nutritional qualities, attractive flavors, and aromas. This has aroused great interest in the export of fruits and their derived products, which makes Brazilian fruit growing a profitable activity in view of the wide diversity of native fruits from the various biomes found in the country [1]. However, the Amazonian fruit species have an agro-industrial potential that has yet to be explored [2,3,4]. Among these, the *bacurizeiro* tree (*Platonia insignis* Mart.) stands out (Figure S1A,B). It is a semi-domesticated fruit tree [5], belonging to the Clusiaceae family and subfamily Clusioideae, and the only species of the monotypic genus *Platonia* Mart. [6]. *P. insignis* originated in the Amazon, with the Brazilian State of Pará considered as its center of diversity, with emphasis on populations located in the mesoregions of Northeast Pará and Ilha do Marajó [5,7]. From Pará, it spread to other states in the northern region, where it is almost always found in areas of primary vegetation, except for the States of Roraima and Tocantins, where it is also present in areas of secondary vegetation. Toward the center-west it only reached Mato Grosso State, and toward the northeast, it reached the States of Piauí and Maranhão, where the *bacurizeiro* populations are present in the transition forest between the Amazon Forest and the Caatinga of the northeastern semi-arid region [8].

The fruit of the *bacurizeiro* tree, called *bacuri* (Figure S1D,E), is rich in amino acids, vitamins, and minerals [9]. The pulp, its main product, is consumed in the form of juices, jellies, ice cream, or raw, and the peel can be used in the preparation of sweets and creams [10]. This species is considered a dual aptitude species because, in addition to the maximum use of each part of the fruit, the wood of the *bacurizeiro* tree, being resistant and large (20–30 m), is intensively used in the construction of boats and houses in many areas of natural occurrence of this species [11]. Due to its economic interest, the species is at great risk of genetic erosion. With clandestine logging, the expansion of the agricultural frontier, and the urban growth of cities, the species is threatened with a loss of genetic variability and even the extinction of populations [12]. Therefore, knowledge about its genetic variability is extremely important, to allow efficient conservation strategies to be outlined and to guarantee subsidies for future genetic improvement programs [13].

Next generation sequencing (NGS) brought several approaches capable of identifying thousands of markers in almost the entire genome of interest, allowing more efficient studies of the diversity and genetic structure of populations. Among the currently available techniques, genotyping-by-sequencing (GBS) stands out for presenting a simplified protocol in relation to other techniques, and for being suitable for population genetic studies, considering that it can be used for any species with a low cost per sample and without the need for a reference genome [14]. The GBS technique enables the identification of thousands of single nucleotide polymorphisms (SNPs), randomly distributed throughout the genome, and the genotyping of populations can be performed on a large scale [14,15]. As current techniques for obtaining SNP markers are efficient and utilize a greater number of markers, it requires a smaller number of accessions per population [16]. This technique allows evolutionary studies to take on a genomic-scale approach [17,18], where conservation-oriented questions can be answered more accurately [19]. More robust and extensive measurements using the large-scale study of the genome make it possible to better analyze genetic variation based on neutral loci and to assess adaptive-based genetic variation. The detection of outlier loci is a strategy to find genetic variation potentially related to local adaptation.

Few studies on population genetics have been carried out so far regarding the *bacurizeiro* tree [1,20,21,22], with most of them using ISSR (Inter Simple Sequence Repeat) markers. Paraense et al. [23] developed 22 microsatellite markers (nSSRs) for *bacurizeiro*. Of these, eight nSSRs, in addition to three chloroplast microsatellites (cpSSRs), were used by Nascimento et al. [24] to evaluate seven populations of the species, originating from two Brazilian biomes (Amazon and Cerrado). Such studies did not elucidate some aspects, such as loci under possible selection effects and the detection of large-scale variability, which can be better understood by population genomics. The present study expanded the sampling of the species carried out by previous studies, using for the first time the GBS technique with NGS technology and obtaining SNP markers. The objectives of the study were the following: to assess the genetic structure and diversity of six sampled locations of *bacurizeiro* trees present in the two main biomes of its occurrence, the Amazon Forest and Cerrado; to highlight useful information for the development of actions for the conservation of the species; and to identify markers (outlier loci) that might be associated with selection and adaptation to different environments. Our hypothesis was that due to anthropic action in the sampled areas, the genetic diversity might be compromised, showing reduced genetic diversity and increased genetic structure among the sampled locations.

## 2. Results

### 2.1. Genetic Diversity and Population Structure of Bacurizeiro

Sequencing of the GBS library resulted in a total of 32.4 million reads. After quality control, demultiplexing, and filtering out the low-quality sequences, 2031 high-quality SNP markers were identified. These markers were used for characterization of the genetic diversity and structure of the 39 bacurizeiro accessions sampled from nine locations in the States of Amazonas, Pará, Rondônia (Amazon biome), and Maranhão (Cerrado biome).

**Figure 1:**
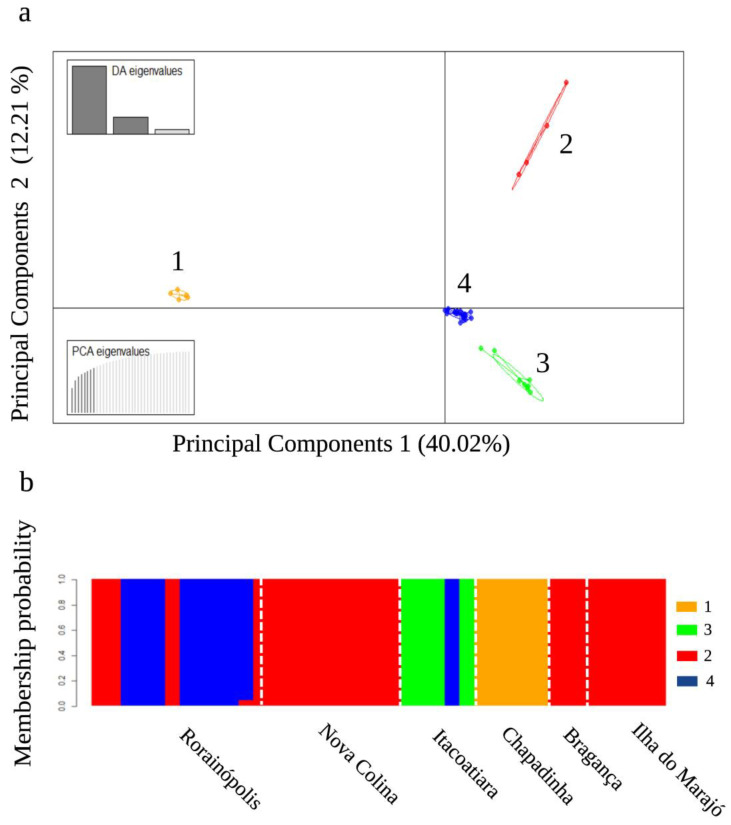
Discriminant Analysis of Principal Components (DAPC) performed based on 2031 SNP markers for 39 bacurizeiro (*Platonia insignis*) accessions: (**a**) scatter plot of clusters formed by the K-means method; and (**b**) clustering probability analysis according to results generated in the DAPC. The dotted lines indicate the studied locations [Rorainópolis, Nova Colina, Itacoatiara, Bragança, Ilha do Marajó (Amazon biome), and Chapadinha (Cerrado biome)].

For the discriminant analysis of principal components (DAPC) analysis, 38 principal components were retained, of which 8 explained 73.2% of the total data variation. The K-means method allowed the identification of four genetic groups with clear differentiation, high structuring, and low admixture (Figure 1a,b; Figure S2). Group 1 was formed exclusively by individuals from the location of Chapadinha, Cerrado biome; this group was genetically distant and isolated from the other groups belonging to the Amazon biome, which are closely related. Group 2 was formed by the individuals from Bragança and Ilha do Marajó, State of Pará, and Nova Colina and Rorainópolis, State of Roraima. A larger portion of the accessions from Rorainópolis formed group 4, including an accession from Itacoatiara, while group 3 was formed mostly by individuals from Itacoatiara.

**Figure 2:**
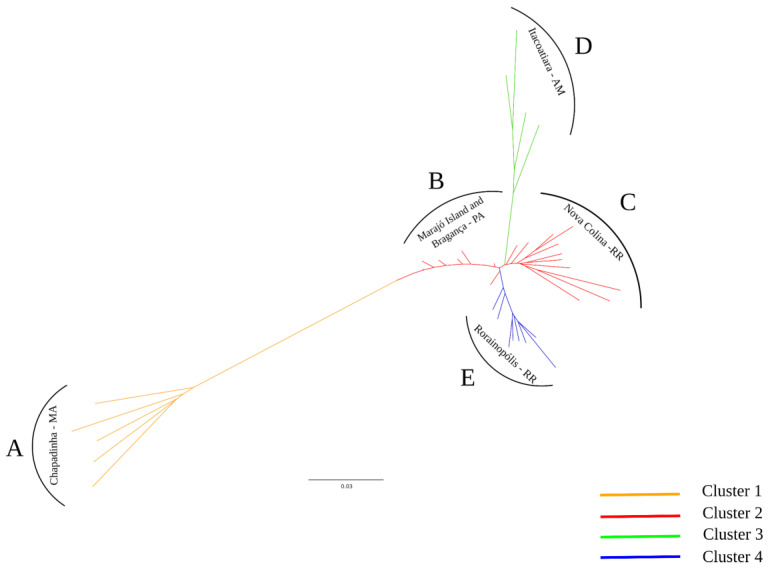
Neighbor-joining tree for 39 samples of *bacurizeiro* (*Platonia insignis*), based on 2031 SNP markers, characterized by the structuring of the groups identified in the discriminant analysis of principal components (DAPC). MA—Maranhão State (Cerrado biome); PA—Pará State; AM—Amazonas State; and RR—Roraima State (Amazon biome).

The neighbor-joining tree grouped the *bacurizeiro* samples into five groups, A–E (Figure 2), which were consistent with the groups defined in the DAPC analysis, with some exceptions. Again, group A, containing accessions from Chapadinha, Cerrado biome, was genetically distant from the accessions of the Amazon biome (groups B–E). Group D, Itacoatiara location, was in an intermediate position in the representation of the dendrogram, between the locations of the States of Pará (group B) and Roraima (group C) (Figure 2), although genetically more distant from them. Rorainópolis (group E), with eight of the nine accessions present in group 4 of the DAPC analysis, is genetically closer to the accessions of Nova Colina, both from the State of Roraima.

The Molecular Variance Analysis (AMOVA) indicated that the genetic variation is greater among than within locations, and greater among than within DAPC groups (Table 1). The associated *F_ST_* estimates for these analyses also suggest that there is high and significant genetic differentiation among locations (*F_ST_* = 0.68) and DAPC groups (*F_ST_* = 0.59). The Mantel test identified a positive and significant correlation (r = 0.85; *p* < 0.001) between geographic distances and genetic divergence, thus showing that the greater the distance between locations, the greater the genetic differentiation between them, indicating the existence of isolation by distance.

The pairwise matrix from the *F_ST_* between locations (Table 2; Figure S3) indicates that the lowest values of genetic differentiation were found between Chapadinha and Bragança, Chapadinha and Ilha do Marajó, and Ilha do Marajó and Bragança. These are the locations with the shortest distance between them. The highest *F_ST_* values were observed among Bragança and Rorainópolis, Ilha do Marajó and Rorainópolis, Bragança and Nova Colina, and Ilha do Marajó and Nova Colina, which are the locations most distant from each other.

Regarding the genetic diversity estimates, the observed heterozygosity for the sampled locations and for the groups defined by the DAPC presented, in most cases, lower values when compared to the expected heterozygosity (Table 3). An excess of heterozygotes indicated by the negative inbreeding coefficients occurred only for Bragança location and groups 1 and 4 from DAPC. However, the accessions from Bragança showed low expected heterozygosity values. Among the DAPC groups, group 1 had the highest genetic diversity values. This group is formed by the location of Chapadinha, in the Cerrado biome (Table 3). Considering the locations, the average *H_E_* value for the Amazon biome locations was much lower (*H_E_* = 0.0750) when compared to the Cerrado biome location (*H_E_* = 0.1746). Chapadinha also had the highest value for the observed heterozygosity (*H_O_* = 0.2078), much higher than the *H_O_* value for the Amazon biome locations (*H_O_* = 0.0558).

The number of alleles among the clusters defined by the DAPC showed similar values, with the lowest value found for group 4 and the highest values for groups 1 and 2 (Table 3). The greatest difference was between biomes, with a much higher value found for the Cerrado biome (*A* = 2981) than the average value for the Amazon biome (*A* = 1697.4). Most of the sampled locations presented a similar number of alleles, except for the two locations from the State of Pará, which showed low values in comparison with the other regions. The number of samples from each location did not interfere with the diversity parameters, as Chapadinha, with only five samples, presented a greater number of alleles, observed heterozygosity, and expected heterozygosity. On the other hand, the locations of the State of Roraima, with a higher number of samples (12 and 9 accessions), showed lower values of these estimates. The fixation index for groups 2 and 3 indicates the occurrence of inbreeding in these two groups (Table 3). Among the locations, Itacoatiara presented the highest fixation index, followed by Rorainópolis and Nova Colina. The locations of Pará and Maranhão showed the lowest fixation indices.

### 2.2. Outlier Loci Analysis

**Figure 3:**
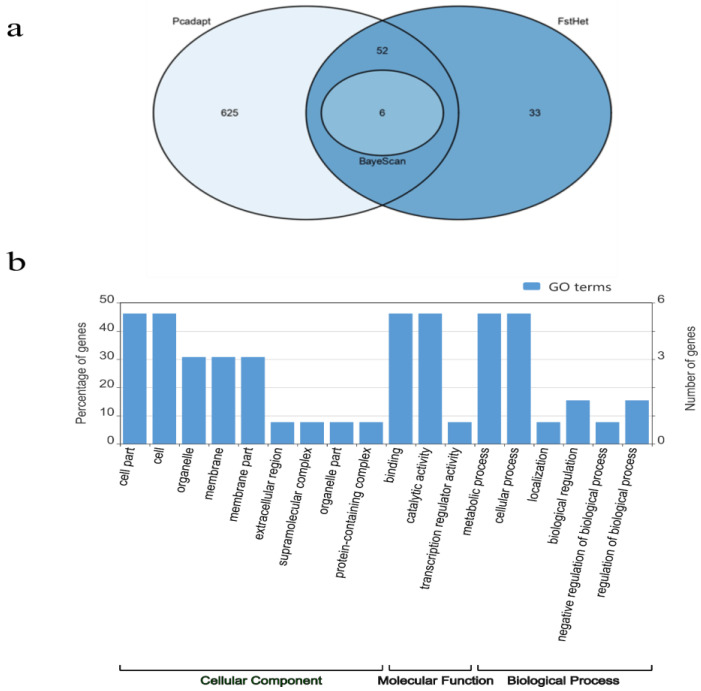
(**a**) Venn diagram showing the number of outlier SNPs detected for each test (in parentheses) and the overlap between them (numbers in ellipses); (**b**) summary of GO terms found among the 58 sequences with outlier SNPs supposedly under selection. The GO terms are grouped according to their biological processes, molecular functions, or cellular components.

A total of 625 outlier SNPs were found in the sampled *bacurizeiro* locations, of which 58 SNPs were identified by at least two of the three methods used, and only 6 were common to the three methods tested (Figure 3a). Among the 58 sequences hypothetically under selection, 13 were similar to annotated genes, presenting GO (Gene Ontology) terms distributed in different classes (Figure 3b). The most frequent GO annotations were associated with molecular functions (10) and biological processes of metabolism (10), as well as cellular components (8), in which the greatest number of sequences were compatible with cell parts, catalytic activity, metabolic processes, and cellular processes (Table S1; Figure 3b). For the blastx analysis, 11 sequences with hits above 53% identity and 55% coverage were returned. The other sequences returned as uncharacterized and hypothetical proteins, as no hits were found in the database.

## 3. Discussion

### 3.1. Genetic Diversity and Structure among the Bacurizeiro Sampled Locations

The groups formed in DAPC and the neighbor-joining tree are consistent with the sampled locations, and the little admixture observed indicates a high genetic differentiation among them. The fact that most of the genetic variation is concentrated among and not within locations (*F_ST_* = 0.68; *p* < 0.001) contradicts the studies published so far for *P. insignis* [20,22,24] and is not expected in the case of allogamous species. It is possible that our results are different because we evaluated very distant locations from different biomes, although a similar study [24] with SSR markers, also including locations from the Amazon and Cerrado biomes, detected that most of the variability was found within locations (72%), and within DAPC groups (78%). Pontes et al. [20] and Pena et al. [22] used ISSR markers to characterize accessions collected in different locations of Pará, including Marajó Island, maintained in the Eastern Amazon BAG (Active Germplasm Bank) [12]. The results showed that genetic variation is greater within than between locations, with low genetic differentiation between them, also contrasting with the present study.

The genetic differences observed in the DAPC clusters, in the phylogenetic tree, and in the *F_ST_* pairwise matrix indicate that the Chapadinha location, which represents a sample from the Cerrado biome, is genetically distant from samples of the Amazon biome, which was also observed by Nascimento et al. [24]. The correlation between genetic and geographic distances confirmed by the Mantel test, and the strong structure between the Amazon and the Cerrado biomes observed in this study and in Nascimento et al. [24] make the isolation of populations by distance evident. Saraiva et al. [25] reported that *P. insignis* populations in different environments that are geographically isolated may accumulate genetic differences, suggesting that the individuals experience habitat-specific selective pressures, the result of which may be ecotypic differentiation, which might explain our results.

The sampled locations in the State of Roraima (Nova Colina and Rorainópolis) are present in secondary vegetation, while Itacoatiara, in the State of Amazonas, occurs in primary vegetation [8]. During the collections, the team found it difficult to find areas where the species occurs in these two states. According to reports from residents, these locations have suffered a reduction in their native vegetation due to fires and the expansion of pasture areas, leading to a reduced number of bacurizeiro trees (Figure S4). The intense exploitation for logging of the *bacurizeiro* trees since the 1970s also indicates the fragility of this species. In the State of Pará, considered a center of diversity for *bacurizeiro* [5,7], its populations are widely recognized by residents and easily found in the main fruit markets. Conversely, in the States of Amazonas and Roraima, *bacurizeiro* fruits are hardly found in markets and are little recognized by the local population. Nova Colina and Rorainópolis, in Roraima, are in the last stage of anthropic action, with the formation of large sandbanks due to the impoverishment of the soil, which in this case is already naturally poor in the Amazon Forest when water bodies that bring nutrients are removed [26]. In Itacoatiara, Amazonas State, the collection site is within the area of the company Precious Woods Mil Madeiras. This company takes low-impact forestry exploration very seriously and does everything it can to always keep its areas preserved. Even though the vegetation in this area is a climax forest, protected by the company mentioned above, the bacurizeiro plants are still rare, meaning that it is really in danger of becoming extinct. However, the fact that our group found bacurizeiros in Itacoatiara is also important, because it represents another new record of the species.

We can suggest from these observations, the DAPC, and the neighbor-joining tree that genetic drift may be occurring, leading to greater differentiation between these locations. This can be confirmed by the highest levels of inbreeding observed for the locations from Roraima and Amazonas. Regarding the estimates of genetic diversity, the locations from the Amazon biome presented low values of diversity, similar to data obtained for an aromatic shrub plant species occurring widely in Northeast Brazil, *Croton tetradenius* (*H_O_* = 0.072; *H_E_* = 0.086) assessed with SNP markers [27], agreeing with our initial hypothesis. The Chapadinha location from the Cerrado biome, on the other hand, presented higher genetic diversity indexes, similar to those obtained for mango trees (*H_O_* = 0.183–0.215; *H_E_* = 0.171–0.216) from seven geographical regions in the world [28]. The reduced genetic diversity of the Amazon biome locations may indicate that these locations are partly formed by clones or related individuals. This situation occurs when individuals have the same maternal origin [29]. Also, the sporophytic self-incompatibility of *P. insignis* [7,30] aggravates this situation. Another reason is that, although the bacurizeiro is currently better known and explored as an edible fruit-producing plant, in the past it was more important as a timber species. Before the arrival of Europeans in Brazil, it was already used by Amazonian indigenous people to build canoes. Nowadays, clandestine logging as well as the expansion of the agricultural frontier are responsible for considerable genetic erosion [12].

The higher diversity indices found in Chapadinha, from the Cerrado biome, were also reported by Nascimento et al. [24], evaluating *bacurizeiro* accessions from the Amazon and Cerrado biomes with microsatellite markers. Chapadinha showed between moderate and high values of genetic diversity and an absence of inbreeding in both studies ([24], present study), indicating the good state of preservation in this area. This occurs due to the presence of an area of environmental protection, Chapada Limpa Extractive Reserve, which contributes to reducing the effects of genetic erosion. However, the moderate values of diversity observed in these areas may be already showing the effects of anthropic action.

It is worth mentioning that in the past, Chapadinha belonged to this protected area and currently only part of its population occurs in the Reserve, which is subjected to urban advance. We know that the use and occupation of protected areas, despite the restrictions, do not prevent irregular activities from taking place [31], such as logging and the opening of swiddens for agriculture. Thus, the populations of *bacurizeiro* trees in Maranhão have been affected by glyphosate, the main herbicide used in soybean plantations (*Glycine max* (L.) Merr.) [32]. The use of this herbicide causes *bacurizeiro* seedlings to not develop normally, leading to the non-renewal of individuals in the location, which can be accentuated by the poor and acidic soils (from sandy to clayey) in this region [33]. These factors may contribute to significant losses of genetic diversity, in addition to preventing the action of soil regeneration carried out by *bacurizeiro* trees [33].

The locations of Bragança and Ilha do Marajó, in the State of Pará, showed low genetic diversity. However, the fixation values showed that both locations do not present inbreeding. Higher inbreeding levels, such as those found for the Amazon locations, are not expected for a predominantly allogamous species with sporophytic self-incompatibility, such as the *bacurizeiro* [7,30]. Studies regarding the pollination of this species are still scarce, with most of them carried out in unnatural conditions. Self-pollination in *P. insignis* is a possibility that should be considered, indicating that this species is in transition to the mixed system [25,34]. The inbreeding observed in this study may also suggest the inefficient action of pollinators, mainly birds [24,35], given the conditions of habitat degradation and the reduction of these visitors to *bacurizeiro* flowers.

The results obtained in this study show that effective measures for the preservation of bacurizeiro populations must be taken urgently. In the study of Nascimento et al. [26] with natural populations from regions similar to ours, important strategies were suggested, including raising awareness about the felling of *bacurizeiro* trees and the early harvest of their fruits. Environmental education programs emphasizing the recognition of fruit species for the local population can help in the awareness process, while adding other measures more focused on public policies can be more effective. The felling of *bacurizeiro* trees for the implementation of pasture areas or cultivation of other species of agronomic interest is extremely problematic not only for the *P. insignis* species but also for its pollinators due to the loss of their habitat [36]. The implementation of agroforestry systems can be an efficient strategy for the recovery of deforested areas and generate subsidies for local populations [37]. In situ conservation in areas of occurrence of bacurizeiro trees would be important, especially in the Amazon biome. Efforts must be made to ensure that *P. insignis* germplasm banks have sufficient genetic variability, as there is clearly a need to increase the representativeness of accessions from populations in the States of Roraima and Amazonas before these populations become extinct.

### 3.2. Outlier Loci

The genotyping of SNP markers obtained in this study allowed the identification of loci with deviations from the expected neutral behavior; that is, those supposedly under selection. The identification of these outlier loci is an important step in understanding local adaptation and evaluating the evolutionary potential of a species [38]. There are no reference genomes for species of the Clusiaceae family. In this case, the search for selection signatures within locations becomes unfeasible as the basis for comparing the GO terms with the database becomes limited. However, this study means a big step toward finding information about these regions, possibly under the effect of selection for a species for which little is known in this regard, contributing to other studies within the family. Most of the found loci are associated with proteins involved in metabolic processes, molecular functions, and, more specifically, catalytic activity and cellular processes. In this sense, it is worth highlighting that some loci showed similarity with the WRKY (Wrinkled, Kluveromyces Yeast), a transcription factor related to the direct activation of expressions that participate in the stress response and plant development processes [39]. Furthermore, both binding and activation of this factor by other types of proteins were shown to be temperature dependent, suggesting that its action resembles a switch from transcriptional repression at normal temperatures to activation at cooling temperatures. The type 2C protein phosphatase family (PP2C) and the sucrose non-fermenting-related protein kinase (SnRK2) subfamily showed similarity to the bacurizeiro outlier loci, which is interesting because they are central players in several stress signaling pathways. In this sense, phosphatases and kinases, in their phosphorylation process for signal transduction, act during responses to abiotic stress [40,41].

One of the hits returned in our analysis was named “probable pyrase 7”. The family of apyrase enzymes is a conserved family of NTDases that can remove the terminal phosphate from NTP (Nucleoside Triphosphate) and NDPs (ribo-Nucleoside Diphosphates). All members of this family share common structural features and participate in different functions within cells, such as the glycosylation of proteins in the Golgi (in yeast, for example) and the regulation of plasma membranes in animals and plants [42,43]. A study using transgenic plants showed that these enzymes, when overexpressed, can aid growth, and develop a more robust architecture for the root system in *Arabidopsis* [44]. Since such an architecture influences water uptake, transgenic plants showed greater tolerance to osmotic stress and water deprivation than wild-type plants. Thus, the constitutive expression of a gene encoding an apyrase results in a better architecture of the root system and, consequently, better survival under water stress conditions. The study also suggested the same mechanism for soybean plants [44].

Another hit found in our blastx analyses, with 93% identity, is a TMK (transmembrane kinase) type receptor kinase. These transmembrane receptor kinases coordinate plant growth and stress responses by regulating acidification or alkalinization, as well as the interspersed pathways of auxin and abscisic acid. However, these proteins participate in several signaling pathways, with emphasis on plant growth balance pathways and stress responses. Thus, when there are changes in the environment, these proteins are phosphorylated or can phosphorylate other components of their pathway to facilitate plant growth and development, as well as assist in stress resistance in the roots [41]. Furthermore, another hit found in the same analysis, with 83.3% identity, is a kinase. In general, in model plants such as *Arabidopsis* and rice, genes encoding kinases and transcription factors are important in activating other genes involved in reactive oxygen species (ROS) toxicity triggered by abiotic stresses [45].

Overall, this study provides valuable information about the molecular mechanisms underlying the stress responses and developmental processes investigated in bacurizeiro populations. These mechanisms indicate potential plant responses to degraded environments with poor soils and high temperatures. Future studies investigating these aspects further could provide deeper insights into the issues raised here.

## 4. Materials and Methods

### 4.1. Sampling, DNA Extraction and Quantification

**Figure 4:**
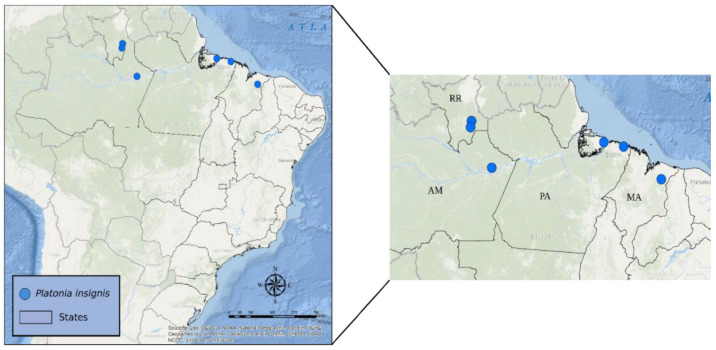
Map with collection sites for *Platonia insignis* accessions in the Brazilian States of Amazonas (AM), Roraima (RR), Pará (PA) (Amazon biome), and Maranhão (MA) (Cerrado biome).

Leaf segments were collected from 39 individuals belonging to six *bacurizeiro* locations distributed among the States of Amazonas (Municipality of Itacoatiara), Pará (Municipalities of Bragança and Ilha do Marajó), Roraima (Municipalities of Rorainópolis and Nova Colina), and Maranhão (Municipality of Chapadinha) (Table 4; Figure 4). This research is registered in the National System for the Management of Genetic Heritage and Associated Traditional Knowledge (SisGen) (registration no A3AF200).

Genomic DNA was extracted from leaves using the Inglis et al. [46] protocol with some modifications, including three to four prewashes using sorbitol wash buffer (100 mM Tris-HCl pH 8.0, 0.35 M Sorbitol, 5 mM EDTA pH 8.0, and 1% (*w*/*v*) Polyvinylpyrrolidone (average molecular weight 40,000; PVP-40)). DNA was resuspended in 25 μL of TE buffer, treated with 0.1 mg mL^−1^ of RNase A, and kept at −20 °C. The quantification and quality analysis of the DNA were performed through electrophoresis in a 1% agarose gel (*w*/*v*) stained with Gel Red. DNA was evaluated for quantity based on the phage λ molecular size standards (Invitrogen, Waltham, MA, USA) at different concentrations (20, 50, and 100 ng μL^−1^) and validated with Qubit 4 fluorometer (Invitrogen). After quantification, DNA samples were normalized to a concentration of 20 ng μL^−1^ for GBS library preparation.

### 4.2. Assembly of the Genomic Library and SNP Identification

The normalized samples, i.e., all containing the same concentration of 30 ng/μL, were initially digested with two restriction enzymes, PstI and MesI, previously tested for bacurizeiro plants following the optimized protocol by Poland et al. [14]. The digested fragments were ligated by the complementary cohesive ends to specific adapter sequences (barcodes) using the NEB T4 DNA ligase enzyme #M0202, the NEB Buffer4 buffer, and the addition of ATP. The ligation reaction products were multiplexed (pooled with all samples identified with a barcode) and subjected to PCR amplification. A library containing 39 samples was obtained by enriching the adapter fragments through polymerase chain reaction (PCR), using Illumina primers with complementary sequences to the adapters. Subsequently, the samples were sequenced in a flow cell of an Illumina HiSeq2500 (Illumina, San Diego, CA, USA) sequencer at the EcoMol Genomics Center at the Luiz de Queiroz College of Agriculture, University of São Paulo.

The discovery and filtering of SNPs were performed using Stacks v. 1.42 [47]. Initially, the filtering involved the quality control step of the sequences from the process_radtags component, where the low-quality sequences were discarded. The sequences of each sample were separated according to the different barcodes (demultiplex), and then the remaining sequences were analyzed using the ustacks component. The function of this component is to identify possible loci present in the genetic material of each sample, with the parameters -m 3, -M 2, and -N 2. The next component used by the program was cstacks, with the -n 2 parameter, responsible for creating a catalog with all the loci identified across individuals. After this step, two components were used to cross-reference information between the loci obtained for each individual and the loci in the catalog (stacks), and to remove the loci with a lower probability (rxstacks, --lnl_lim -10). The population component was used for the final filtering of SNP markers. Only one SNP per tag was retained, with sequencing depth ≥ 3x MAF ≥ 0.01; SNP present in at least 60% of samples within populations; SNP present in at least 3 of the 4 (bacuri) states sampled.

### 4.3. Identification of Outlier Loci

The identification of markers under selection pressure called outliers was performed based on tests that considered the sample groups by state. Three complementary tests were performed: Pcadapt [48], in which the outlier loci are associated with the genetic groups observed in a principal component analysis (PCA); fsthet [49], for identifying loci with excessively high or low *F_ST_* values relative to a neutral distribution; and BayeScan [50], a Bayesian analysis for estimating posterior probabilities to verify whether each locus reflects selection. The pcadapt analysis was performed with the first two principal components, where SNP markers with q-values < 0.1 were considered outliers. The fsthet analysis was performed based on the betahat estimate [51] (analogous to the *F_ST_*), considering as outliers the SNP markers above or below a 95% confidence interval constructed based on 1000 bootstraps. The above analyses were performed with the R program packages [52], pcadapt [48], and fsthet [49]. The BayeScan 2.1 program [50] was used to perform 20 pilot runs with 100,000 iterations each, followed by 250,000 burn-in steps and 25,000 steps with intervals of 50 (total of 1,500,000 iterations). The probability of including selection was 3 times lower than not including selection in the model. In this analysis, SNP markers with FDR < 0.05 were considered outliers.

The occurrence of false positives is frequent in the detection of outlier loci [53]. For this reason, the final set of outlier markers consisted of the loci identified in at least two of the three applied tests, as suggested by Luikart et al. [53]. The similarity of sequences with outlier markers with proteins annotated in Genbank was evaluated with the blast2GO program [54], using the blastx algorithm. Possible functional annotations associated with the proteins were summarized using the Gene Ontology (GO) terms (http://geneontology.org/, accessed on 22 September 2022) with the online tool WEGO (http://wego.genomics.cn/, accessed on 22 September 2022). In addition, the Uniprot database (https://www.uniprot.org, accessed on 22 September 2022) was used to better understand the functions of the proteins returned as best hits for the outlier sequences. Likewise, the sequences were submitted to the InterPro platform (https://www.ebi.ac.uk/interpro, accessed on 22 September 2022) to find possible domains.

### 4.4. Statistical Analyses

All the statistical analyses were performed in R software version 4.3.3 [52]. The discriminant analysis of principal components (DAPC) was performed with the adegenet package [55]. The number of clusters from the DAPC was calculated using the K-means method, which runs different probabilities of cluster numbers. Among the probability models generated by the K-means method, the Bayesian Information Criterion (BIC) method was considered to determine the number and nature of the groups.

The genetic relationship between the 39 accessions of *bacurizeiro* trees was analyzed by the cluster analysis obtained from the neighbor-joining method and genetic distances of Nei [56], using the ape package [57]. Using the FigTree v.1.4.3 program (http://tree.bio.ed.ac.uk/software/Figtree/, accessed on 30 November 2021), the dendrogram was edited. The pairwise *F_ST_* matrix based on the evaluated locations was calculated from the poppr package [58] and visualized using the corrplot v. 0.84 package [59].

The genetic diversity parameters, such as the total number of alleles (*A*), the observed (*H_O_*) and expected (*H_E_*) heterozygosity, and the Wright’s inbreeding coefficient (*f*) [60], were estimated according to the groups delimited by DAPC and for the evaluated locations with the hierfstat package [61] and poppr [58]. The distribution of genetic variability between and within locations, as well as between and within the groups defined by the DAPC, was estimated using AMOVA, with hierfstat [61] and poppr [58,62]. To verify whether isolation by distance was occurring, the Mantel test was performed with the aid of the ade4 package [63,64,65,66,67], aiming to evaluate the correlation between the genetic divergence from the *F_ST_* values of the pairwise matrix between locations and the geographic distance, generated from the geographic coordinates and built with the help of the geodist package [68].

## 5. Conclusions

The genetic analysis of bacurizeiro populations revealed complex patterns of genetic structure and variation among locations, diverging from previous studies. The genetic disparities between locations within the Amazon and Cerrado biomes, particularly the higher genetic diversity observed in Chapadinha within the Cerrado biome and lower diversity found in the Amazon biome locations, highlight the detrimental impacts of human activities on the genetic diversity of these populations. The presence of inbreeding and potential genetic bottlenecks emphasizes the urgency of conservation actions to protect the genetic diversity of the bacurizeiro. Functional analysis of outlier loci provided valuable insights into essential genes, underscoring the importance of preserving genetic diversity for species adaptation and survival. Therefore, it is crucial to implement effective conservation measures, raise public awareness, and adopt sustainable practices to ensure the protection and continuity of the genetic resources of bacurizeiro trees, safeguarding their ecological and economic relevance for future generations.

## Figures and Tables

**Table 1:** Molecular analysis of variance (AMOVA) for *Platonia insignis* based on 2031 SNP markers performed for the assessed locations, and the discriminant analysis of principal components (DAPC) groups.

Source of Variation	DF	Sum of Square	Coefficient of Variation	Variation Percentage (%)	F Statistics
Among locations	3	15,987.13	478.761	68.34	*F_ST_ =* 0.68 *
Within locations	33	7317.50	221.742	31.65	
Among DAPC groups	3	3373.08	126.89	59.79	*F_ST_ =* 0.59 *
Within DAPC groups	35	2986.95	85.341	40.21	

DF = degrees of freedom. * Significant at *p* < 0.05 (*p*-values = 5 × 10^−5^).

**Table 2:** A pairwise matrix of geographical distances between the sampled locations ^1^ of *Platonia insignis*, on the upper diagonal, and on the lower diagonal, the *F_ST_* values calculated between the locations. The displayed distance values are in kilometers.

Sampled Locations	RO	NC	IT	BR	IM	CH
RO		57.3	471.3	1537.3	1335.1	1967.1
NC	0.28		425.0	1538.3	1336.7	1960.8
IT	0.33	0.29		1330.6	1142.3	1693.7
BR	0.71	0.69	0.48		202.4	478.6
IM	0.69	0.68	0.55	0.16		665.4
CH	0.62	0.60	0.55	0.04	0.11	

^1^ RO—Rorainópolis and NC—Nova Colina, Roraima; IT—Itacoatiara, Amazonas; BR—Bragança and IM—Ilha do Marajó, Pará (Amazon biome); and CH—Chapadinha, Maranhão (Cerrado biome).

**Table 3:** Genetic diversity estimates based on 2031 SNP markers evaluated in 39 samples of *Platonia insignis*, considering the groups identified by the discriminant analysis of principal components (DAPC) and the sampled locations.

Group	*N* ^1^	*A*	*H_O_*	*H_E_*	*f*
1	5	2981	0.2078	0.1746	−0.1904
2	21	2764	0.0561	0.0983	0.4295
3	4	2339	0.0562	0.0832	0.3242
4	9	2184	0.0461	0.0435	−0.0585
Mean		2567	0.0915	0.0999	0.1262
Locations ^2^/Amazon biome					
RO	12	2310	0.0493	0.0665	0.2589
NC	9	2326	0.0563	0.0752	0.2512
IT	5	2566	0.0519	0.1262	0.5885
BR	3	609	0.0572	0.0424	−0.3500
IM	5	676	0.0644	0.0649	0.0071
Mean		1697.4	0.0558	0.0750	0.1511
Cerrado biome					
CH	5	2981	0.2078	0.1746	0.1904

^1^ Number of accessions (*N*), total allele number (*A*), observed heterozygosity (*H_O_*), expected heterozygosity (*H_E_*), and Wright inbreeding coefficient (*f*). ^2^ RO—Rorainópolis and NC—Nova Colina, Roraima; IT—Itacoatiara, Amazonas; BR—Bragança and IM—Ilha do Marajó, Pará; and CH—Chapadinha, Maranhão.

**Table 4:** Locations of *Platonia insignis* accessions collected, with details of the number of individuals sampled, biome, and geographic coordinates.

Location	Municipality/ State	Number of Individuals	Biome	Latitude	Longitude
IT	Itacoatiara/AM	5	Amazon	02°47′47.0″ S	58°34′15.1″ W
BR	Bragança/PA	3	Amazon	01°03′27.5″ S	46°44′9.91″ W
IM	Ilha do Marajó/PA	5	Amazon	00°41′37.0″ S	48°31′07.0″ W
RO	Rorainópolis/RR	12	Amazon	01°03′14.7″ N	60°23′10.4″ W
NC	Nova Colina/RR	9	Amazon	00°32′29.9″ N	60°27′50.5″ W
CH	Chapadinha/MA	5	Cerrado	03°44′30.0″ S	43°21′37.0″ W
Total		39			

## Data Availability

The datasets generated and analyzed during the current study are available in the Mendeley repository: https://data.mendeley.com/datasets/rwvdfkghkn/1, published on 15 September 2023.

## Supplementary Materials

The following supporting information can be downloaded at https://www.mdpi.com/article/10.3390/plants13071033/s1, Figure S1: (A) *bacurizeiro* tree (*Platonia insignis*) from the population of Chapadinha in the State of Maranhão, Cerrado biome; (B) *bacurizeiro* tree from the population of Itacoatiara in the State of Amazonas, Amazon biome; (C) flowers from the *bacurizeiro* tree, source: author’s photo collection; (D) bacuri fruit; and (E) ripe bacuri fruit, source: [69]. Figure S2: PCcadapt results for detection of discrepant SNPs considering *bacurizeiro* tree (*Platonia insignis*) groups by state and biomes [AM, PA, and RR from Amazon biome, and MA from Cerrado biome]. Analyzes were performed based on 2031 SNP markers. (a) Scree plots of the proportion of variance explained in principal component analysis (PCA) for the first K = 20 principal components. The number of components retained in the analyzes followed Cattle’s rule, choosing the point on the left where the curve bends (K = 4), after which the addition of components does not substantially increase the amount of explained variance; and (b) Scatter plot of the first two principal components showing the main genetic structure observed in the PCAs for the sample clusters. Figure S3: Pairwise matrix based on the genetic differentiation values (*F_ST_*) among the 39 analyzed *Platonia insignis* samples grouped by location. Figure S4: Satellite images obtained from Google Earth on the left (available at: http://earth.google.com/, accessed on 15 December 2021) and photos taken by the collection team for the present study at the locality of Nova Colina, Roraima State, Amazon biome. Table S1: Outlier loci obtained for *Platonia insignis* locations.
