# Supplementary material for: Low Diversity and High Genetic Structure for Platonia insignis Mart., an Endangered Fruit Tree Species
Source: Plants (Basel). 2024 Apr 6;13(7):1033. doi: 10.3390/plants13071033 (PMC11013813; doi:10.3390/plants13071033)
Supplement: Supplementary file 1 [file plants-13-01033-s001.zip › Supplementary Material Figures S1–S4.pdf]

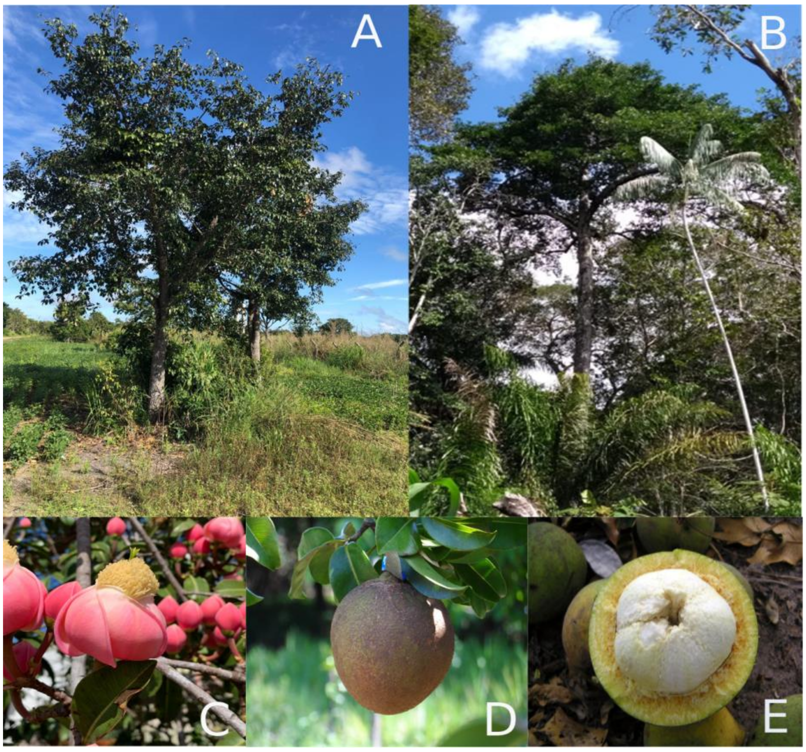

Figure S1. (A) *Bacurizeiro* tree (*Platonia insignis*). from the population of Chapadinha in the State of Maranhão, Cerrado biome; (B) Bacurizeiro tree from the population of Itacoatiara in the State of Amazonas, Amazon biome; (C) Flowers from the *bacurizeiro* tree. Source: author´s photo collection; (D) Bacuri fruit; (E) Ripe bacuri fruit. Source [69].

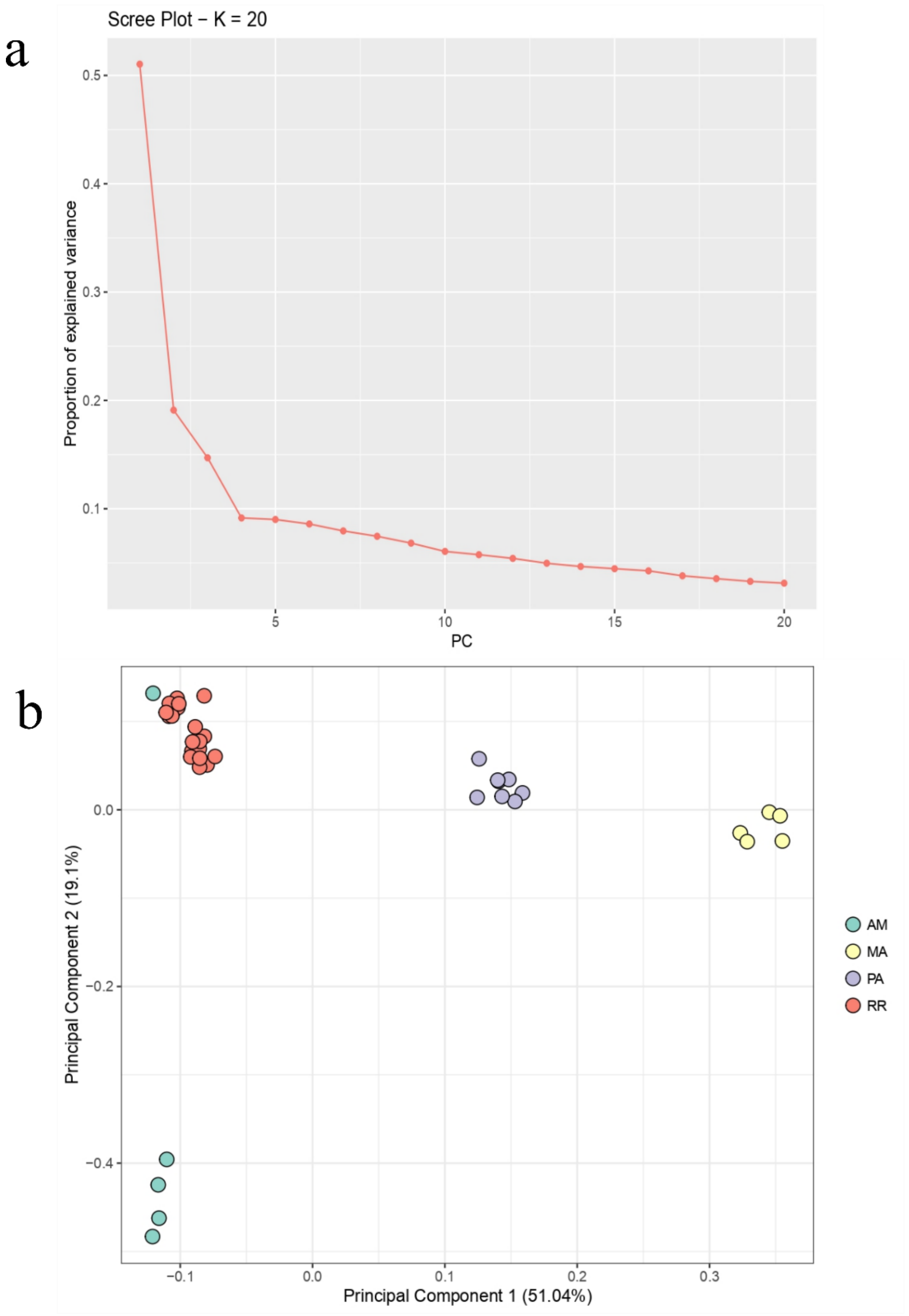

Figure S2. PCcadapt results for detection of discrepant SNPs considering *bacurizeiro* tree (*Platonia insignis*) groups by State and biomes [AM, PA, RR from Amazon biome, and MA from Cerrado biome]. Analyzes were performed based on 2,031 SNP markers. a) Scree plots of the proportion of variance explained in principal component analysis (PCA)

for the first K = 20 principal components. The number of components retained in the analyzes followed Cattle's rule, choosing the point on the left where the curve bends (K = 4), after which the addition of components does not substantially increase the amount of explained variance; b) Scatter plot of the first two principal components showing the main genetic structure observed in the PCAs for the sample clusters.

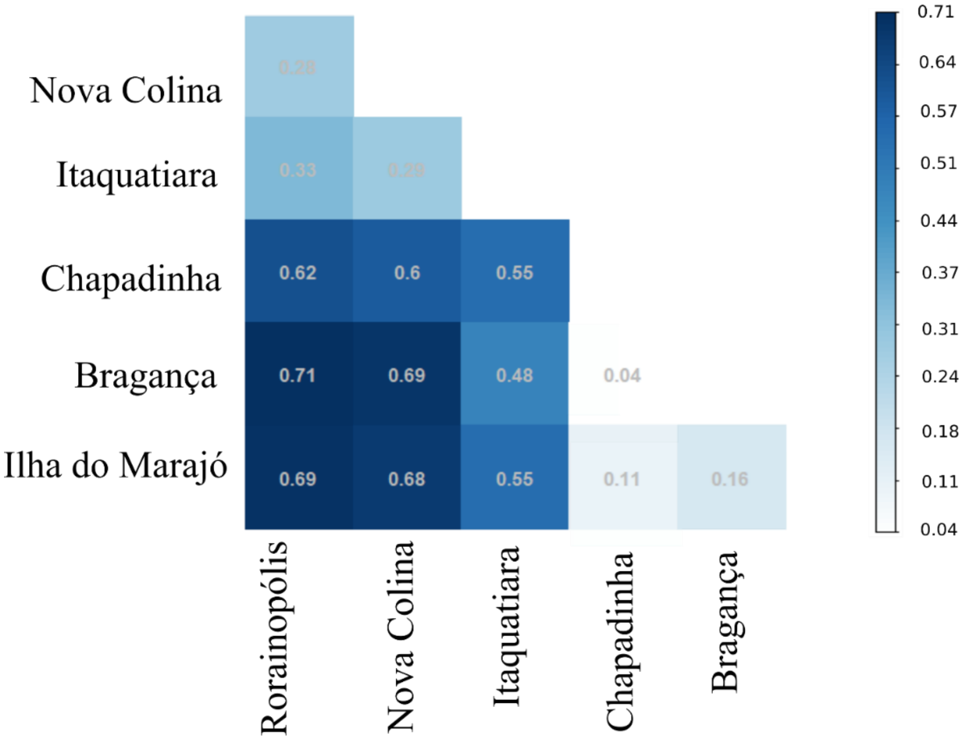

Figure S3. Pairwise matrix based on the genetic differentiation values (F_{ST}) among the 39 analyzed *Platonia insignis* samples grouped by location.

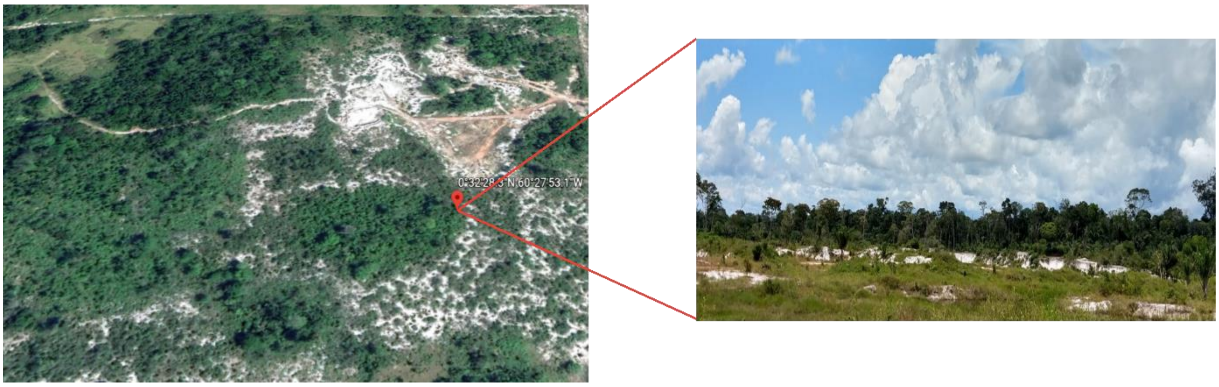

Figure S4. Satellite images obtained from Google Earth on the left (available at http://earth.google.com/, 2021) and photos taken by the collection team for the present study at the locality of Nova Colina, Roraima State, Amazon biome.
