# Supplementary material for: Low Diversity and High Genetic Structure for Platonia insignis Mart., an Endangered Fruit Tree Species
Source: Plants (Basel). 2024 Apr 6;13(7):1033. doi: 10.3390/plants13071033 (PMC11013813; doi:10.3390/plants13071033)
Supplement: Supplementary file 1 [file plants-13-01033-s001.zip › Supplementary Material Table S1.pdf]

Table S1. Outlier loci obtained for *Platonia insignis* samples.

ID	Sequences	Blastx	% identity	% cover	Uniprot	Function	Interpro
CLocus _385	TGCAGCTCCAATTGCAGACAT TCCTTTGGCTTTCAAGGCAGA AATAACCTGTTTGGCATCTTC ATATCCTTTGTCCTACCAAGG AATCAAAT	Endo-1,3;1,4-beta-D-glucanase-like	80%	81%	Q6ZJQ2 (rice)	Hydrolase activity (group of enzymes that catalyze the breaking of covalent bonds in reaction with water)	none predicted
CLocus _3725	TGCAGGTGCTGTAGGAGGTG TTGCAGCTTCTCTTATTCGTG TTCCTACAGAGGTTTATTTGC CTTTTCTAGCTAGAAGTATGA GCTTTGATT	S-adenosylmethionine carrier 1, chloroplastic/mitochondrial	94.44%	58%	A3BIT2_ORYSJ (rice)	Integral component of the membrane, transmembrane transport	none predicted
CLocus _4575	TGCAGTAGTGACTTCTAGCAT CCATGTAGCTGGGTTGTATCC ATCTTTGATTTTAGGGACTCC TTGAATTCCCTTCAAAAGAGA AAGATGCC	Pleiotropic drug resistance protein 1-like isoform X1	72.41%	94%	A0A6P5ZSC7_DURZI (durian)	Integral component of the membrane, transport activity, ATP binding	none predicted
CLocus _6950	TGCAGGTAACATCAGTAAAT GACATGTACAAACTTTTACAA GAGTACAATGCTAGCTTGCA GCTGTACAATAGTAAACTTCA ACAAGACCTC	kinesin-like protein KIN-14N isoform X3	83.33%	97%	A0A8B8NG63_9MYRT (*Rhodamnia argentea*)	ATP binding, binding/movement/ microtubule motor activity, cytoskeletal motor activity	none predicted
CLocus _7172	TGCAGCTGTGAAACTAGGAT CTCTAGCCAAGGAAGAAGCC ATTTGTTGGATGAATATCTGT TCAGTTGAGGATGATTTGCTT TCAAGCAGCA	Probable WRKY transcription factor 60	53.57%	91%	A0A8B8NNR4_9MYRT (*Rhodamnia argentea*)	DNA binding, transcription factor activity	none predicted
CLocus _9256	TGCAGTTGGGCTACAAGGAT CAATATGTGTGTTCACGATGT GCTTCGAGTCAAAAAGATGG TATTAGTCCCGCCATTGGAGC AAGAACTTCG	Probable apyrase 7	53.33%	97%	Q6Z4P2 · APY2_ORYSJ (rice)	Catalyzes hydrolysis of phosphoanhydride bonds of nucleoside tri- and diphosphates, nucleotide binding, hydrolase activity, integral component of membrane	none predicted
CLocus _12465	TGCAGCTAAATGGCCCATCTG ACCACATAGAAAGCATTTAT CTTGCTGTCCAGGAGTGAAA ACAACCTGAATGATAACATC AAAGGGCAGAT	5'-3' exoribonuclease 3-like isoform X1	78.57%	91%	A0A1S4BJW1_TOBAC - protein RRP6-like 3 isoform X1 (*Nicotiana tabacum* (Common tobacco)	Nucleotide and RNA binding, exoribonuclease activity, integral membrane component, nuclear exosome, histone mRNA catabolic process, nuclear polyadenylation	none predicted

Clocus _16070	TGCAGGTCCAGTGATGTCATAACTTCCAAGCCCTATCAAGTAATTGGAAGCAGAGAGAACCATTCCACCATTGAAAAGAAACAGTAGCAAAT	Neutral ceramidase 1-like	73.33%	97%	A0A7J0EBD9_9ERIC (kiwi)	Ceramidase activity (enzyme that cleaves fatty acids from ceramide, producing sphingosine which in turn is phosphorylated by a sphingosine kinase to form sphingosine-1-phosphate), kinase, phosphorylation, hydrolase, transferase	none predicted
CLocus _18131	TGCAGGTACTCTCCCTTCAGACCTAAGGAACCTTACTCAACTAGAGAGGTTAGAGCTCCAATGGAACAATATCTCTGGCCCTTTGCCTAGTT	Receptor protein kinase TMK1-like isoform X1	93.10%	94%	A0A6P5T5E6 · A0A6P5T5E6_PRUAV - (Cherry) (*Cerasus avium)*	Integral component of membrane, ATP binding, kinase activity, protein phosphorylation	none predicted
CLocus _18216	TGCAGTTCACAAGGAACTAAAGTAACGTTACCTCGAGACCTGAAAGAGCATTCCAAGAAGACAAAGGTTCAACGGCAGAATCATCGATGAGA	Protein phosphatase 1 regulatory subunit pprA	75.00%	65%	A0A654FLL7 · A0A654FLL7_ARATH - *Arabidopsis*	Not found	none predicted
CLocus _18499	TGCAGGTTTCCAAGCTTTATGAGGTTGTGCCTCCAGTTCTTCATGAGCTGGGCAAGGCATGTGCATTTTCTTACTGGAGCGTAAGGTATGCC	Low Quality Protein: citrate synthase, mitochondrial-like	88.24%	55%	P20115 · CISY4_ARATH	Citrate synthase is found in almost all cells capable of oxidative metabolism. A	none predicted
